# Clinical utilisation of implantable loop recorders in adults with Fabry disease—a multi-centre snapshot study

**DOI:** 10.3389/fcvm.2023.1323214

**Published:** 2023-12-08

**Authors:** Ashwin Roy, Ravi Vijapurapu, Hibba Kurdi, Christopher Orsborne, Peter Woolfson, Manish Kalla, Ana Jovanovic, Christopher A. Miller, James C. Moon, Derralynn A. Hughes, Tarekegn Geberhiwot, Richard P. Steeds

**Affiliations:** ^1^ Institute of Cardiovascular Sciences, University of Birmingham, Birmingham, United Kingdom; ^2^ Department of Cardiology, Queen Elizabeth Hospital Birmingham, University Hospitals Birmingham NHS Foundation Trust, Birmingham, United Kingdom; ^3^ Department of Cardiology, Bart Heart Centre, Barts Health NHS Trust, London, United Kingdom; ^4^ Lysosomal Storage Disorder Unit, Royal London NHS Foundation Trust, University College London, London, United Kingdom; ^5^ Division of Cardiovascular Sciences, University of Manchester, Manchester, United Kingdom; ^6^ Wythenshawe Hospital, Manchester University NHS Foundation Trust, Manchester, United Kingdom; ^7^ Department of Cardiology, Northern Care Alliance NHS Foundation Trust, Salford, United Kingdom; ^8^ Department of Metabolic Medicine, Northern Care Alliance NHS Foundation Trust, Salford, United Kingdom; ^9^ Department of Inherited Metabolic Diseases, Queen Elizabeth Hospital Birmingham, University Hospitals Birmingham NHS Foundation Trust, Birmingham, United Kingdom; ^10^ Institute of Metabolism and Systems Research, University of Birmingham, Birmingham, United Kingdom

**Keywords:** Fabry disease, arrhythmia, stroke, implantable loop recorder, sudden death

## Abstract

Fabry disease (FD) is an X-linked deficiency of alpha-galactosidase-A, leading to lysosomal storage of sphingolipids in multiple organs. Myocardial accumulation contributes to arrhythmia and sudden death, the most common cause of FD mortality. Therefore, there is a need for risk stratification and prediction to target device therapy. Implantable loop recorders (ILRs) allow for continual rhythm monitoring for up to 3 years. Here, we performed a retrospective study to evaluate current ILR utilisation in FD and quantify the burden of arrhythmia that was detected, which resulted in a modification of therapy. This was a snapshot assessment of 915 patients with FD across three specialist centres in England during the period between 1 January 2000 and 1 September 2022. In total, 22 (2.4%) patients underwent clinically indicated ILR implantation. The mean implantation age was 50 years and 13 (59%) patients were female. Following implantation, nine (41%) patients underwent arrhythmia detection, requiring intervention (six on ILR and three post-ILR battery depletion). Three patients experienced sustained atrial high-rate episodes and were started on anticoagulation. Three had non-sustained tachyarrhythmia and were started on beta blockers. Post-ILR battery depletion, one suffered complete heart block and two had sustained ventricular tachycardia, all requiring device therapy. Those with arrhythmia had a shorter PR interval on electrocardiography. This study demonstrates that ILR implantation in FD uncovers a high burden of arrhythmia. ILRs are likely to be underutilised in this pro-arrhythmic cohort, perhaps restricted to those with advanced FD cardiomyopathy. Following battery depletion in three patients as mentioned above, greater vigilance and arrhythmia surveillance are advised for those experiencing major arrhythmic events post-ILR monitoring. Further work is required to establish who would benefit most from implantation.

## Introduction

Fabry disease (FD) is an X-linked lysosomal storage disorder manifesting in multi-organ accumulation of sphingolipids, such as globotriaosylceramide (Gb3), resulting in cardiac, renal, and cerebral manifestations (1). Sphingolipids accumulate because of alpha-galactosidase-A enzyme deficiency (2). Alpha-galactosidase-A is responsible for the breakdown of terminal galactose from Gb3, resulting in lysosomal accumulation of Gb3 (1). Cardiac sphingolipid accumulation occurs in all cardiac cell types, resulting in left ventricular hypertrophy (LVH), myocardial inflammation, fibrosis, and scarring (3). These effects contribute to the high prevalence of arrhythmia in FD, and it is noteworthy that sudden cardiac death (SCD) is the most common form of FD mortality (4, 5). Studies indicate that the rate of frequency of malignant ventricular arrhythmia may be as high as 30% (6). Atrial fibrillation (AF), bradyarrhythmia, ventricular tachyarrhythmia, and SCD may indeed be the first manifestations of cardiomyopathy before the occurrence of clinical or imaging abnormalities (6). However, in published literature, it is shown that the true prevalence of arrhythmia is dependent on both the stage of the disease and the duration of monitoring, with high rates detected on implantable loop recorders (ILRs) in individuals with advanced cardiomyopathy (7). The rates of atrial high-rate episodes (AHREs) detected on implanted cardiac devices are also high (8). This is associated with a higher prevalence of stroke events (9). However, the benefits of anticoagulation for AHREs in the absence of electrocardiogram (ECG) evidence in the general population are outweighed by bleeding risk (10).

To date, research into arrhythmia in FD has been limited to single-centre studies using 12-lead ECG or short-term (<48 h) Holter monitoring, with no evidence from large multi-centre collaborative studies. Current guidelines in the general population recommend ILR implantation in patients who suffer recurrent unexplained symptoms potentially related to arrhythmia after initial assessment that includes patient history, examination, ECG, and transthoracic echocardiography (TTE) (11). Although there are no specific guidelines for treating FD, ILRs have the added benefit of long-term, continual rhythm monitoring for up to 3 years. This may be particularly advantageous in FD cardiomyopathy, which is insidious. Given the potential benefit of ILRs as a tool for arrhythmia detection in FD, we performed a multi-centre retrospective clinical cross-sectional study on the use of ILRs in FD. Our aims were as follows:

1. To provide a comprehensive evaluation of the clinical utilisation of ILRs in FD and
2. To quantify the detected arrhythmia burden, which resulted in a modification or initiation of therapy.

## Methods

This was an observational, retrospective cross-sectional snapshot review of cardiovascular data collected from adults over 18 years of age with genetic or enzymatic confirmation of FD who were referred to three large UK centres managing adults with FD (Queen Elizabeth Hospital, Birmingham; Salford Royal Hospital, Salford; and Royal Free Hospital, London). These centres offer a one-stop service whereby patients undergo detailed clinical assessment including 12-lead ECG, TTE, and cardiac magnetic resonance (CMR) imaging, according to current guidelines (11). All patients were screened between 1 January 2000 and 1 September 2022, and those with prior or current ILRs were included in the analysis. Data were extracted from investigations performed within the preceding 12 months.

This study was approved by local clinical governance committees (CARMS-13350) and it conformed to the principles of Good Clinical Practice guidelines. Ethical approval was obtained for the conduct of this study (IRAS 325613 23/WM/0180). The inclusion and exclusion criteria are detailed below.

Inclusion criteria were as follows:

1. Adults over 18 years of age.
2. Genetic or enzymatic confirmation of FD.
3. History of prior or current ILR implantation on clinical grounds.

Exclusion criteria were as follows:

1. ILR implantation on research grounds.

### Electrocardiography

Standard 12-lead ECGs were acquired according to current standardised guidelines for acquisition and interpretation (12).

### Transthoracic echocardiography

TTE (iE33/EPIC, Phillips; and Vivid, GE) was performed by an accredited sonographer in accordance with the minimum data set guideline of the British Society of Echocardiography (13). The chamber size and function were measured according to current standard guidelines (14). Parameters for assessment of diastolic function were acquired according to the general principles for TTE assessment established by the American Society for Echocardiography in association with the European Association of Cardiovascular Imaging (15). Diastolic function was graded by an experienced cardiologist specialising in TTE according to current guidelines (15).

### Cardiac magnetic resonance imaging

Contrast-enhanced CMR (1.5 T Avanto, Siemens Healthcare, Erlangen, Germany) was performed in line with standard protocols to obtain left ventricular (LV) dimensions, volumes, and mass (16). A steady-state free precision single breath-hold modified Looker Locker inversion recovery (MOLLI) sequence was used for T1 mapping in the basal and mid LV short-axis levels and horizontal long axis, before and 15–20 min after administration of the gadolinium-based contrast agent (17). Imaging for assessment of late gadolinium enhancement (LGE), regional and global T1, and myocardial extracellular volume was performed as previously described (18).

### Statistical analysis

The baseline demographics of the cohort were summarised, with continuous variables reported as means ± standard deviations (SDs). Where two continuous variables were being compared, normality was assessed using the Shapiro–Wilk test. Where data were normally distributed, an unpaired *t*-test was performed. When comparing the relationships between two quantitative and independent variables, a simple linear regression analysis was performed. A *p*-value < 0.05 was deemed to be indicative of statistical significance throughout. All analyses were performed using GraphPad Prism version 9.3.1, GraphPad Software, San Diego, CA, USA (www.graphpad.com).

## Results

**Figure 1:**
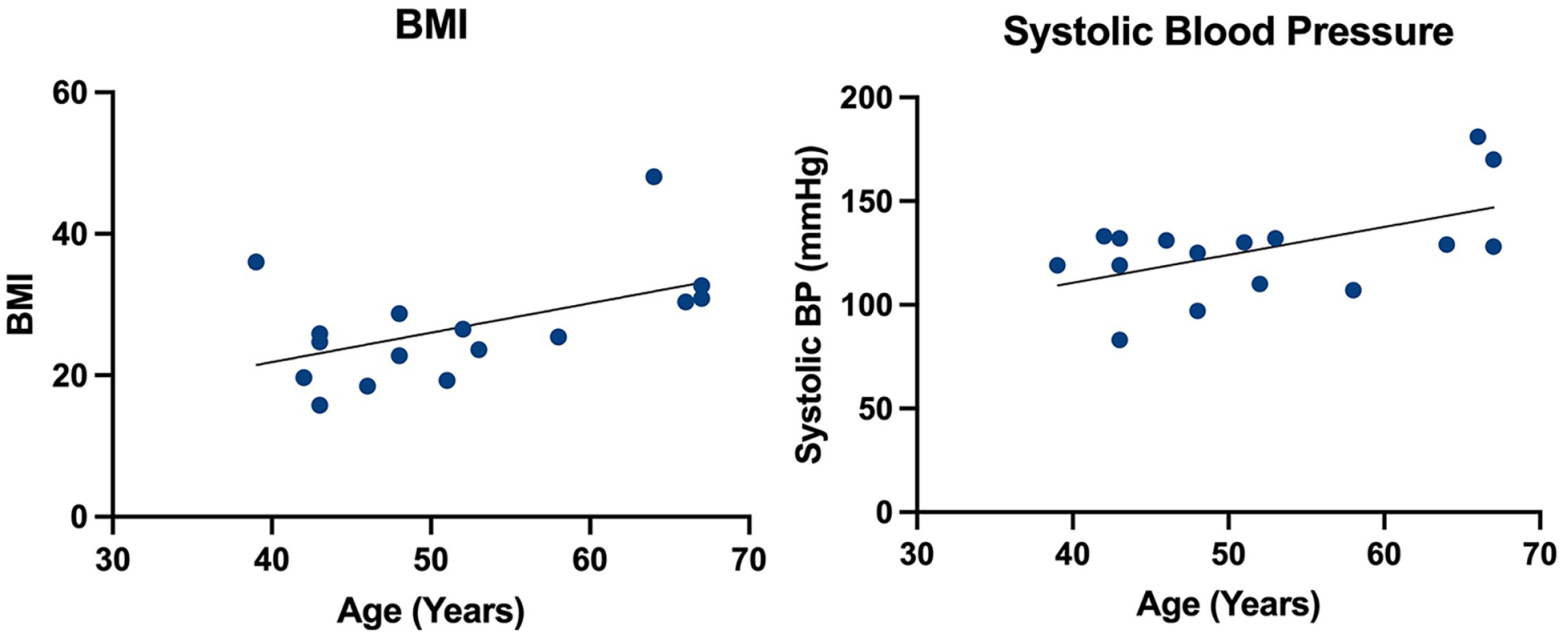
Simple linear regression of age vs. BMI and systolic blood pressure.

In this snapshot assessment, 915 patients with FD across three specialist treatment centres in England were identified between 1 January 2000 and 1 September 2022. Of these, 22 (2.4%) patients underwent clinically indicated ILR implantation. The mean age at the time of implantation was 49.6 ± 9.9 years and 13 (59%) patients were female. The mean systolic blood pressure (BP) level was 127 ± 24 mmHg and diastolic BP level was 75 ± 13 mmHg. The mean body mass index (BMI) was 27 ± 8 kg/m^2^. BMI and systolic BP significantly increased with age (*p* = 0.0434 and *p* = 0.0287, respectively) as illustrated in Figure 1. At the time of implantation, six (27%) patients experienced hypertension, three (14%) had renal impairment defined at an estimated glomerular filtration rate <90 ml/min, six (27%) had a prior cerebrovascular accident (CVA), and 18 (82%) were on enzyme replacement therapy (ERT). Cohort characteristics are described in Table 1.

**Table 1:** Cohort characteristics.

Variable	Number	Mean	SD
Demographics			
Age at implant	22	49.60	9.920
HR (bpm)	16	72.40	11.000
Systolic BP (mmHg)	16	127.00	23.900
Diastolic BP (mmHg)	16	74.90	12.600
Height (m)	21	1.68	0.085
Weight (kg)	21	75.50	18.700
BMI	16	26.80	7.900
Electrocardiogram			
PR interval (ms)	21	149.00	21.800
QRSd (ms)	22	105.00	20.300
Cardiac magnetic resonance imaging			
LVMi (g/m^2^)	13	123.00	53.500
MWT (mm)	16	18.20	5.860
LVEDVi (ml/m^2^)	16	67.90	16.200
LVESVi (ml/m^2^)	16	20.20	8.020
LVSVi (ml/m^2^)	15	37.40	18.300
LVEF (%)	18	70.40	5.280
Septal T1 (ms)	13	898.00	98.800
Transthoracic echocardiogram			
LVEF (%)	20	64.20	6.110
MWT (mm)	19	15.60	3.750
LA volume (ml)	17	44.10	17.800

LVSVi, indexed left ventricular stroke volume.

At the time of implantation, 21 (96%) patients had a sinus rhythm and one patient had AF. The mean heart rate, PR interval, QRS duration, and QTc were in the normal range. Altogether, 21 (95%) patients had TTE imaging data available. The mean left ventricular ejection fraction (LVEF) was in the upper/normal range at 64% ± 6% and the mean maximum wall thickness (MWT) was elevated at 16 ± 18 mm. The mean left atrial (LA) volume was in the normal range at 44 ± 18 ml. Among the patients whose diastolic function could be assessed, 14 (67%) had evidence of diastolic dysfunction.

Eighteen (82%) patients had CMR imaging data available. On CMR, 16 (89%) patients had evidence of LVH, with the mean indexed left ventricular mass (LVMi) elevated at 123 ± 54 g/m^2^ with an MWT of 18 ± 6 mm. The mean indexed left ventricular end-diastolic volume (LVEDVi) was 68 ± 16 ml/m^2^ and indexed left ventricular end-systolic volume (LVESVi) was 20 ± 8 ml/m^2^. The mean LVEF was supranormal at 70% ± 5%. Nine (50%) patients had reduced septal T1 relaxation times, indicative of sphingolipid accumulation with a mean septal T1 of 898 ± 99 ms. Fourteen (78%) patients had evidence of LGE, indicative of myocardial fibrosis.

Indications for ILR implantation varied. Twelve (55%) patients underwent implantation for recurrent and persistent symptoms despite normal ECG and Holter monitoring. Of these, eight patients had palpitations and four reported syncopal episodes. Five (23%) were asymptomatic but underwent implantation to seek undiagnosed AF as an aetiology following cryptogenic CVA. Four (18%) were asymptomatic but underwent implantation because of abnormal Holter monitoring [one non-sustained ventricular tachycardia (VT) under 5 s, two sinus pauses between 1 and 2 s, and one AF detection under 10 s]. None of the arrhythmias were detected by Holter-mandated therapy. These patients were all deemed high risk for arrhythmia with evidence of LVH and LGE on CMR with a broad QRSd on ECG. One (4%) patient was asymptomatic with a normal Holter monitor result but underwent ILR implantation and was deemed high risk for arrhythmia with LVH and LGE on CMR with a broad QRSd on ECG.

Following ILR implantation, arrhythmia was detected in nine (41%) patients, who required the initiation of or a change in therapy. Of these nine, six episodes of arrhythmia were detected on ILRs and three were detected within 1 year following ILR battery depletion. Of these patients, one was in the group of four with documented arrhythmia on Holter monitoring; the remaining three did not develop arrhythmia on ILRs or post-ILRs. Of the six patients with arrhythmia on ILRs, three had recurrent and prolonged AHREs for over 6 hours. Given the duration and frequency of these AHREs, they were started on anticoagulation therapy. None of these patients had documented arrhythmia prior to ILR implantation. One had a high burden of symptomatic ventricular ectopy (11%) and was initiated on a beta blocker. This patient had non-sustained VT on Holter prior to implantation. Two had symptomatic non-sustained VT and were also started on beta blockers. Of the three with arrhythmia that occurred 1 year after ILR battery depletion, one was admitted with acute syncope and was found to have prolonged pauses and complete heart block, requiring isoprenaline. The three subsequently underwent dual-chamber implantable cardiac defibrillator (ICD) implantation. Two were admitted with syncope and were found to have bursts of sustained VT on inpatient telemetry. Both these patients underwent dual-chamber ICD implantation. None of the three patients with documented arrhythmia post-ILRs showed evidence of arrhythmia prior to Holter monitoring.

**Figure 2:**
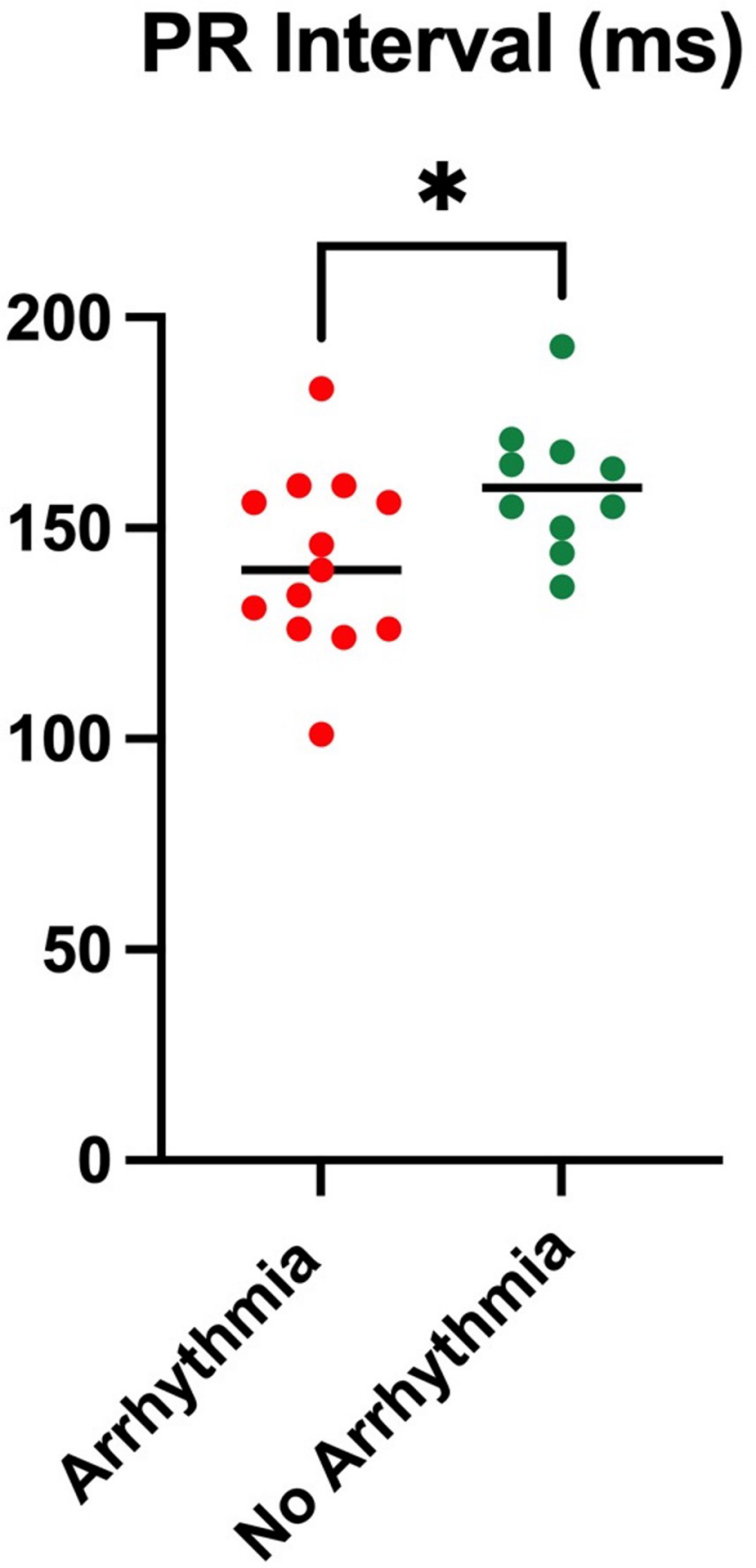
PR interval comparing an arrhythmia cohort with a non-arrhythmia cohort.

By comparing the characteristics in the arrhythmia cohort vs. those without, it was found that those in the arrhythmia group had a significantly shorter PR interval (although still normal) on 12-lead ECG than those without (*p* = 0.0402), as illustrated in Figure 2, with no other ECG changes. Otherwise, no significant differences were observed between the two groups.

## Discussion

This multi-centre snapshot assessment is the first of its kind to report the clinical utilisation of ILRs in FD. We demonstrated that the current UK practice of ILR implantation in FD uncovers a higher-than-expected burden of arrhythmia, requiring initiation or modification of therapy. Our findings are in keeping with those from a previously published single-centre study in adults with advanced FD cardiomyopathy, who demonstrated a high burden of arrhythmia on ILR but no arrhythmia on Holter monitoring (7). However, the patients in that study were included on the basis of advanced disease and did not undergo ILR implantation on clinical grounds. The burden of arrhythmia identified in this study was higher than observed in a previous single and multi-centre study in adults without FD who underwent ILR implantation. In the single-centre study population (post-cryptogenic CVA with no history of arrhythmia), no patients with ILRs implanted developed arrhythmia, and in the multi-centre study population (aged 70–90 years with at least one additional CVA risk factor but no history of CVA), 31.8% developed arrhythmia (19, 20).

Our findings suggest that patients undergoing ILR implantation on clinical grounds have advanced disease, which is evidenced by an increased indexed left ventricular mass, MWT, and supranormal LVEF, and the majority of them have evidence of LGE on CMR indicative of myocardial fibrosis. Unsurprisingly, because of this, the arrhythmia detection rate is high, as these factors are shown to predispose to tachy- and bradyarrhythmia in FD (6, 21). There were no differences in mass, MWT, LVEF, and presence of LGE in those with or without arrhythmia, and this largely reflects the heterogeneity of the cohort, with the majority of patients already having advanced disease at the time of ILR implantation. Furthermore, we also demonstrated that the BMI of patients in this cohort increases with age. The effects of elevated BMI and obesity alter cardiac hemodynamics and probably contribute to the development of LVH in this cohort through systemic inflammation, insulin resistance, and subsequent cardiac remodelling, termed obesity cardiomyopathy (22).

ILRs allow for continual recording and can record episodes of AHRE, which we have demonstrated. As these may resemble AF, clinicians may initiate anticoagulation therapy in the presence of risk factors for stroke (9). This is particularly relevant in FD where the risk of stroke is greater than in the general population. However, a recent large multi-centre study was conducted comparing anticoagulation vs. no anticoagulation in adults with AHRE and at least one risk factor for stroke. This study of 2,536 patients demonstrated that anticoagulation resulted in a higher rate of major bleeding and death compared with placebo, and did not significantly reduce the incidence of stroke, systemic embolisation, and cardiovascular death (10). These findings suggest that even if additional risk factors are present, as is the case in FD, anticoagulation for AHREs without ECG confirmation of AF may not reduce stroke prevalence but rather cause an additional risk of bleeding. At the time when these data were collected and decisions were made about therapy, it was thought that the risk of anticoagulation was outweighed by its potential benefit given the frequency of AHREs (10). A recently published study has highlighted that this may not be the case. Therefore, it is unclear whether these patients should or should not be anticoagulated.

Published outcome data suggest that cardiac symptoms are reported to be more than 60% in men and 45% in women, with symptoms including angina, palpitations, dyspnoea, and syncope (23). Within our cohort, we report a disproportionately low rate of ILR implantation, given the high prevalence of cardiac symptoms reported in FD. With the frequency of symptoms in the FD population and the known risk of arrhythmia, ILR implantation seems to be overly restricted to those with the most advanced stages of FD cardiomyopathy.

PR interval changes are commonly reported in FD, and PQ interval shortening may indeed be one of the earliest features of cardiac involvement. Changes in PQ interval and *P*-wave duration are presumed to be secondary to atrial sphingolipid deposition, which may take place prior to the onset of LVH (24). We demonstrated that in those with arrhythmia detected on ILRs, the PR interval was shorter, although still within the normal range, compared with the arrhythmia-free cohort. This may suggest that the effects of sphingolipid accumulation alter the conduction pathways between the sinoatrial and the atrioventricular nodes, which has been demonstrated previously (25).

Interestingly, after 3 years of ILR monitoring, a proportion of the cohort subsequently suffered major adverse arrhythmic events, all of whom subsequently required dual-chamber ICD therapy. None of the patients underwent repeat ILR implantation following battery depletion. Among those who underwent Holter monitoring post-ILRs, none of them demonstrated arrhythmia. This highlights the fact that despite the effectiveness of a 3-year continual cardiac rhythm monitoring with ILRs, vigilance is advised in patients with FD. In line with local guidance, patients with FD should continue to undergo frequent ambulatory monitoring, especially given the progressive nature of FD cardiomyopathy with the subsequent arrhythmic risk increasing with age and disease progression (6).

## Limitations

Given the fact that FD is a rare disease, the inevitable limitation of this study is its relatively small sample size, despite the pooling of data from three of the UK’s largest centres for managing patients with FD. Although the number of patients analysed was small, this largely reflects the low utilisation of ILRs, as the total of 915 patients represents one of the largest adult cohorts available in FD. It is also an accepted fact that centres have various reporting criteria and different parameters for the investigations conducted, which can limit the pooling of data for comparison, particularly with T1 mapping on CMR.

Four patients underwent ILR implantation with previously documented arrhythmia on Holter monitoring. They underwent implantation for prolonged monitoring to assist with decision-making regarding therapeutic cardiac device therapy and/or further medical therapy. These patients will be pre-selected for an arrhythmia substrate and therefore are more likely to experience arrhythmia compared to those without documented arrhythmia prior to ILR implantation.

It is also important to note that three patients developed arrhythmia post-ILR battery depletion. This suggests that although ILRs allow for prolonged continuous screening, arrhythmia may still be a case of missed diagnosis. A select group of patients with high arrhythmic risk may be considered for re-implantation of ILRs following battery depletion.

## Conclusion

This study demonstrates a high arrhythmia burden detected in adults with FD using ILRs compared to the non-Fabry population, which is in keeping with published literature. ILR implantation is undertaken in patients with advanced FD cardiomyopathy at a stage when their arrhythmic risk is high, and therefore, it is probable that ILRs will be implanted on them at a later stage. ILRs allow for prolonged monitoring and should be considered at an early stage in FD to detect ventricular arrhythmia, conduction disease, and AF. This may reduce the prevalence of SCD and stroke in FD. However, a large-scale study is needed in this area to understand the subgroup of patients who would benefit most from ILR implantation.

## Data Availability

The original contributions presented in the study are included in the article/Supplementary Material; further inquiries can be directed to the corresponding author.
